# A new fluorescent technique for pesticide detection by using metal coordination polymer and nanozyme

**DOI:** 10.1186/s13020-020-00304-2

**Published:** 2020-03-10

**Authors:** Jinchao Wei, Yan Xue, Jiayi Dong, Shuangpeng Wang, Hao Hu, Hao Gao, Peng Li, Yitao Wang

**Affiliations:** 1 Institute of Traditional Chinese Medicine & Natural Products, College of Pharmacy/Guangdong Province Key Laboratory of Pharmacodynamic Constituents of TCM and New Drugs Research, Jinan University, Guangzhou, China; 2 State Key Laboratory of Quality Research in Chinese Medicine, Institute of Chinese Medical Sciences, University of Macau, Macau, China; 3 Institute of Applied Physics and Materials Engineering, University of Macau, Macau, China

**Keywords:** Nanozyme, Organophosphorus, Pesticide, Organophosphorus hydrolase, Para-nitrophenol, Cerium oxide, Methyl-paraoxon, Fluorescent, Polymer, ATP

## Abstract

**Background:**

Chinese herbs have been widely used for thousands of years. In order to kill or control insects and fungus during the cultivation of herb plants, pesticides have played a significant role. More than 30 kinds of pesticides have been documented in the latest version of Chinese Pharmacopoeia. It is urgent to develop new analytical methods for pesticide detection.

**Methods:**

A fluorescent detection system was established by using Cerium based fluorescent polymer and Sm-CeO_2_. As a new doped nanozyme, Sm-CeO_2_ exhibits OPH-like activity to hydrolyze OPP pesticide.

**Results:**

The morphology of the prepared CFP and Sm-CeO_2_ were characterized. The optimal conditions for CFP synthesis are CeCl_3_ (16 mmol L^−1^, 200 μL), ATP (4 mmol L^−1^, 200 μL) and Tris buffer (5 mmol L^−1^, 500 μL, pH 8.0). Sm-CeO_2_ shows the best mimic activity to hydrolyze OPP pesticide at pH = 8.0. The results exhibit good linear relationship between fluorescent quenching effect and MP in the range of 2–50 μmol L^−1^. Finally, this fluorescent technique was successfully applied in *Poria cocos* and Semen Coicis sample.

**Conclusions:**

It is the first report on OPP detection by using CFP and doped nanozyme. The successful application in real sample indicates this method is a rapid, reliable strategy to detect OPP in Chinese herbs.

## Background

Chinese herbal plants have been widely used as health care products, dietary supplements, and therapeutic drugs. For instance, *curcuma longa* and its extract are shown to treat several malignant diseases [1]. Clinical evidence and extensive studies showed that berberine as an effective agent for treating infectious diarrhea and has significant antibacterial and antipyretic effects [2]. As the main active component of honeysuckle to antioxidant and anti-inflammatory, chlorogenic acid could protect against high-fat diet-induced oxidative stress and mitochondrial dysfunction in liver [3]. In the process of industrialization of herbal plants, pesticides have played a significant role with their proven effectiveness of killing or controlling unwanted insects, fungus and pets. The combined use of multiple pesticides is very common in the actual planting process. In the latest version of the Chinese Pharmacopoeia published in 2019, the number and types of pesticides residues have risen significantly [4]. Up to now, 33 pesticides have been restricted in the use of traditional Chinese herbs, including organochlorine pesticide, organophosphorus pesticide, carbamate, herbicide, etc. And among them, organophosphorus pesticide (OPP) has been listed as a new and important type of pesticides. OPP has been proven to be effective and efficient in crops planting, as well as plant diseases and insects’ control. Consequently, OPP has been found to be the major contaminant in water, environment and eco-chain [5, 6]. In the pollution situation of traditional Chinese medicinal materials, OPP is the main object of pesticide pollution [7–10]. Inevitably, the highly prevalent use of pesticides has made the quality and safety of the herbal products a highly worrisome problem. The contaminated herbs may cause poisoning, severe illness, cancers, and other forms of serious health problem. Therefore, to establish new analytical methods for OPP detection is the key to guarantee the safety in use of Chinese herb plants.

Instrumental analysis is the mainstream strategy documented in the Chinese Pharmacopoeia as the golden standard. Coupled with sample preparation procedure, the analytical instruments including gas chromatography, liquid chromatography, mass spectrometer were often used for pesticide residue detection in Chinese herbs with their high sensitivity and high accuracy [11–13]. In the meanwhile, newly developed biosensing methods have been emerging in recent years, which have been an inseparable part in the pesticide detection [14, 15]. Organophosphorus hydrolase (OPH) is a very useful enzyme in disposing and detecting OPPs with high specificity [16, 17]. However, the difficulty and price of extracting OPH is high while the stability of OPH is quite low. In our previous research, we have synthesized nanoceria as OPH-like nanozyme and establish a fluorescence detection system [18]. In the current research, we will explore the usage of another doped nanozyme “Sm-CeO_2_” with mimic activity.

In this study, a novel fluorescent method has been established on the basis of doped nanozyme and metal coordination polymer for pesticide detection. Featuring with large stokes shifts and narrow emission bands, metal coordination polymers have been widely used [19, 20]. The metal coordination polymers in this work were assembled from Ce^3+^ with adenosine triphosphate (ATP). And ATP serves as organic ligands to sensitize the luminescence of Ce^3+^. As mentioned before, most of the sensing techniques are developed based on OPH enzyme, the limitations on application include poor stability, demanding storage conditions and high cost. In the present study, we explored the potential application of Sm-CeO_2_ nanoparticle as a new doped nanozyme with OPH-like activity. Furthermore, the applicability of the proposed detection system was investigated in a real sample. To the best of our knowledge, the fluorescent assay for detecting pesticide by using doped nanozyme has been reported rarely.

## Methods

### Reagents and materials

Cerium chloride (CeCl_3_), Methyl-paraoxon (MP) and Samarium doped cerium oxide (Sm-CeO_2_) were obtained from Sigma-Aldrich (USA). Adenosine triphosphate (ATP) and Para-nitrophenol (p-NP) were purchased from J&K Scientific Ltd. (China). Tris–HCl buffer and ultrapure water (Millipore, ≥ 18 MΩ cm) were used throughout the experiments.

### Instruments

The size and morphology of Cerium based fluorescent polymer (CFP) were characterized by field emission scanning electron microscopy (FESEM, Zeiss Sigma, Germany). High-resolution transmission electron microscopy (HRTEM) images were obtained by employing Talos F200X operating with 200 kV electron beams (FEI, Thermo Fisher Scientific, USA). Sm-CeO_2_ samples were dispersed in ethanol and depositing droplets on Cu grids with the solvent evaporated under ambient conditions. Fluorescence spectra were performed on a Lumina fluorescence spectrometer (Thermo Fisher Scientific, USA). UV–vis absorption spectra were recorded by DR 6000 UV–vis spectrophotometer (HACH, USA). All experiments were carried out at room temperature.

### Synthesis and preparation of CFP

The CFP were prepared according to the previous study with some modifications [19, 21]. In brief, water (350 µL), CeCl_3_ (16 mmol L^−1^, 200 μL), and ATP (4 mmol L^−1^, 200 μL) were added gradually into Tris–HCl buffer (5 mmol L^−1^, 500 μL, pH 8.0) to form a white flocculent suspension under stirring at room temperature.

### Detection of methyl-paraoxon

Sm-CeO_2_ (10 mg) was first added into the 550 μL Tris–HCl buffer solution (pH 8.0, 5 mmol L^−1^). After the addition of 50 μL sample solution containing different concentrations of MP, the tube was centrifuged (10,000*g*, 5 min) to obtain the supernatants. Thus, 500 μL supernatants was transferred into 2.0 mL centrifugal tube with the successive addition of water, CeCl_3_ and ATP. The fluorescence spectra were monitored in the emission wavelength range of 320–700 nm while the excitation wavelength was 310 nm. The fluorescence (FL) intensity was measured at wavelength of 350 nm and 615 nm.

### Sample preparation

Dried *Poria cocos* and semen coicis sample was bought from a local supermarket. The samples were weighted and extracted with ethyl acetate (1 mL g^−1^) for 10 min. The supernatants (10.0 mL) was obtained after the centrifugation (4550 rpm, 5 min), and evaporated to dryness under a stream of nitrogen. The residue was re-dissolved with Tris buffer (1.0 mL) for analysis.

## Results

### Characterization of CFP and Sm-CeO_2_

**Fig. 1:**
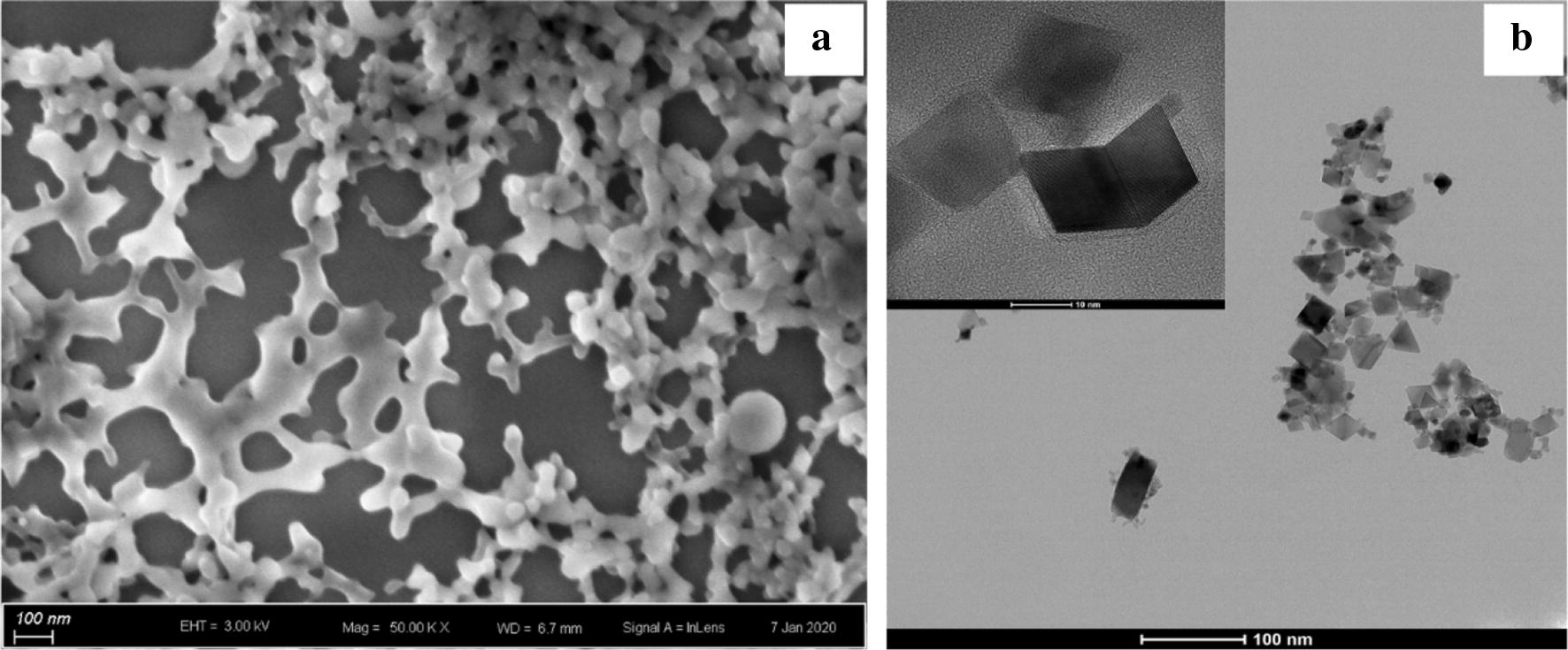
**a** SEM image of the prepared CFP sample. **b** HRTEM and TEM images of Sm-CeO_2_ sample

**Fig. 2:**
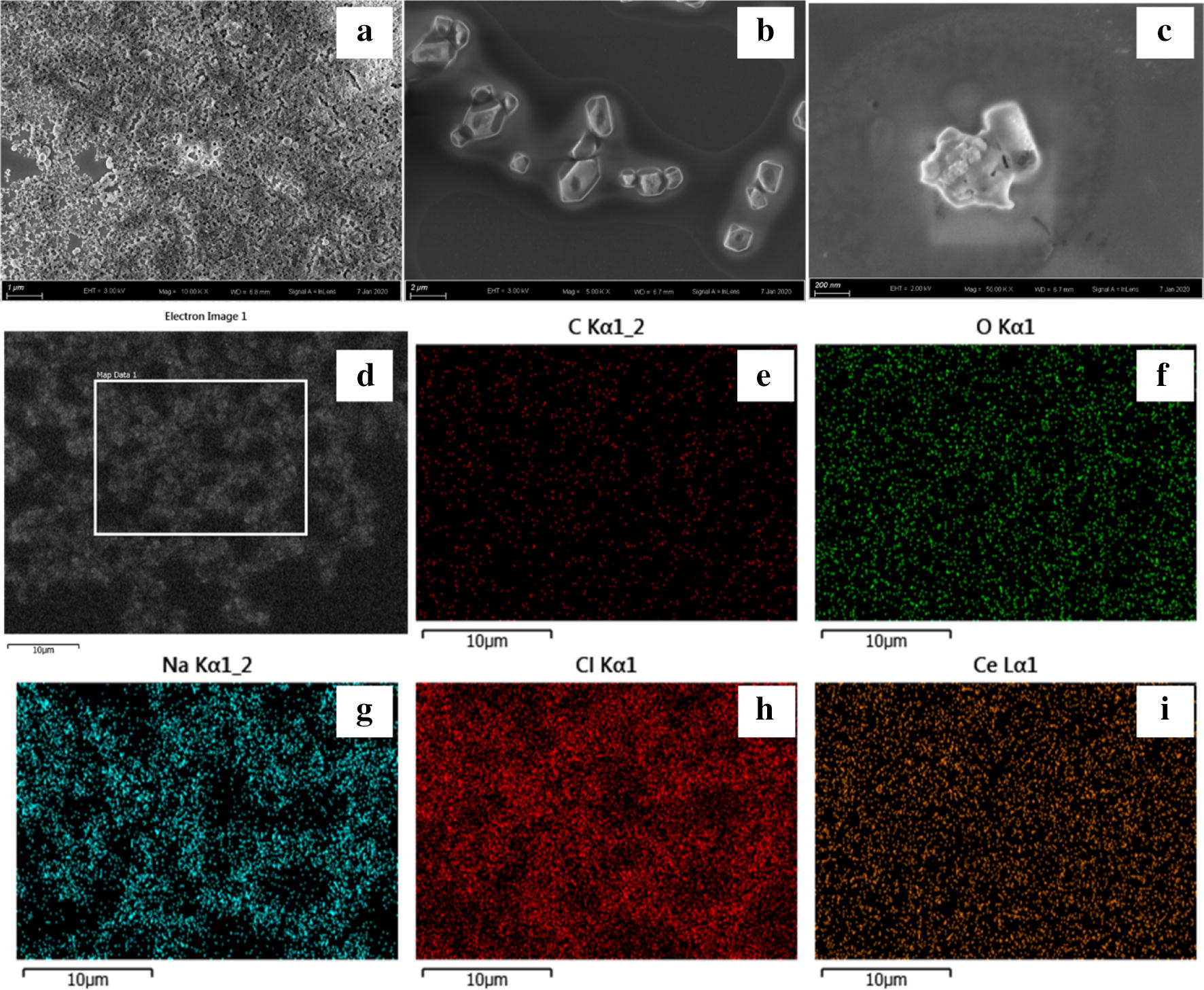
SEM images of the CFP (**a**), ATP-Ce (**b**), and Tris-Ce (**c**) sample and images of EDX elemental mapping for CFP sample (**d**, image of EDX scan area. **e**, C; **f**, O; **g**, Na; **h**, Cl; **i**, Ce)

**Fig. 3:**
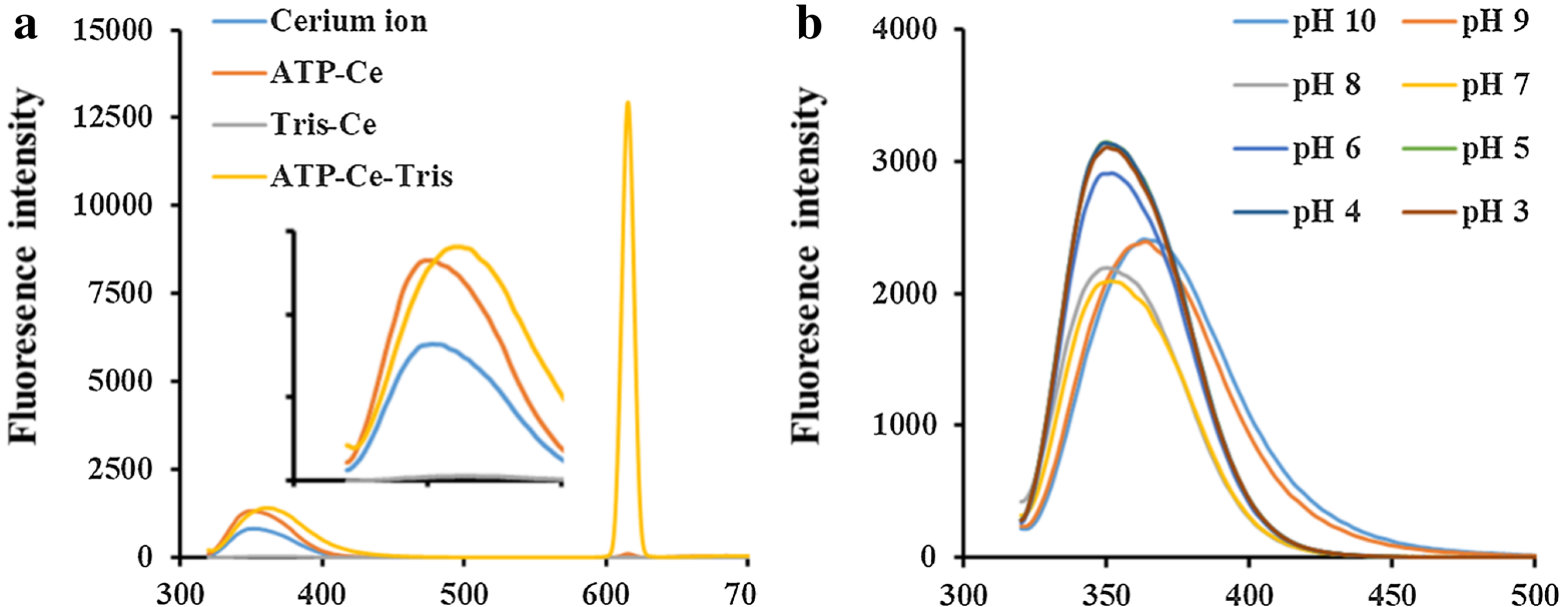
**a** FL spectra of cerium chloride solution (blue line), ATP-Ce solution (brown line), Tris-Ce solution (grey line), and the CFP solution (yellow line). **b** The effect of pH from 3 to 10 on the FL spectra of CFP. (Excitation wavelength at 310 nm)

The morphology and size of prepared polymer were first analyzed by scanning electron microscopy. The polymers were spheres with diameter about 50 nm (Fig. 1a). The morphology of Sm-CeO_2_ was characterized by HRTEM in Fig. 1b. They were cubes or polyhedral with an average diameter around 10 nm. Along with the fabrication of CFP sample, we also synthesized ATP-Ce and Tris-Ce samples to compare their significant differences on the morphologies (Fig. 2a–c). Furthermore, EDX analysis confirmed the presence of carbon, oxygen, cerium and other atoms on the surface of the CFP samples (Fig. 2d–i). Then the as-synthesized polymers were optically characterized by using fluorescence spectra. The fluorescence emission spectrum peaked at 350–370 nm and 615 nm upon excitation at 310 nm (Fig. 3a). As an extremely good chelator, ATP with triphosphate chain can coordinate to metal ions to form a macrochelate due to the high affinity between phosphate groups and metal ions [22, 23]. The emission peak of cerium nitrate aqueous solution was located at 350 nm. As expected, the FL intensity was enhanced with the addition of ATP to cerium solution, which suggested that ATP-Ce has been obtained (Fig. 3a inset). Tris molecule act as a cofactor ligand to prepare the CFPs and enhance the luminescence [21]. As presented in Fig. 3a, the FL intensity at 615 nm was dramatically increased with the addition of both ATP and Tris accompanied by the emission peak at 350 nm red shift to 370 nm, which confirmed the formation of CFPs.

### Optimization of CFP

**Fig. 4:**
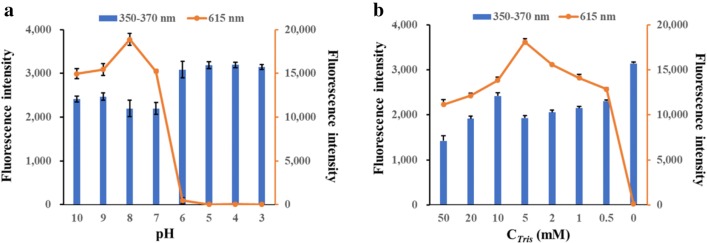
**a** FL intensities of CFP under different pH conditions. **b** The effect of Tris concentration on the FL intensity of CFP samples

To improve the fluorescence performance of the polymers, pH value and Tris concentration were optimized in this study. In our work, the effect of pH ranged from 3 to 10 on the fluorescence was presented in Figs. 3b and 4a while Tris concentration was kept at 5 mmol L^−1^. The maximum FL intensity of CFP samples will change from 350 nm to 370 nm due to the pH changes. Then we selected the maximum FL intensity and names as “FL intensity at 350–380 nm” to compare the differences between the CFP samples. The FL intensity at 350-370 nm increased along with peak blueshift when the solution changed from alkaline (pH 7 to 10) to acidic (pH 3 to 6) (Fig. 3b). Furthermore, FL peak at 615 nm decreased dramatically at acidic condition. The white flocculent suspension changes to transparent solution, which suggest the CFP was not exist anymore. Based on this, the optimal pH at 8 was selected when the FL intensity at 350–370 nm and 615 nm were both reached a higher level. The optimal Tris concentration was investigated successively while the pH values were kept at 8. As presented in Fig. 4b, the FL intensities at both two wavelengths were changed with the increasing Tris concentration from 0 to 50 mmol L^−1^. Finally, the optimal concentration at 5 mmol L^−1^ was fixed in further experiments.

## Discussion

### The pH effect on the mimic activity of Sm-CeO_2_

**Fig. 5:**
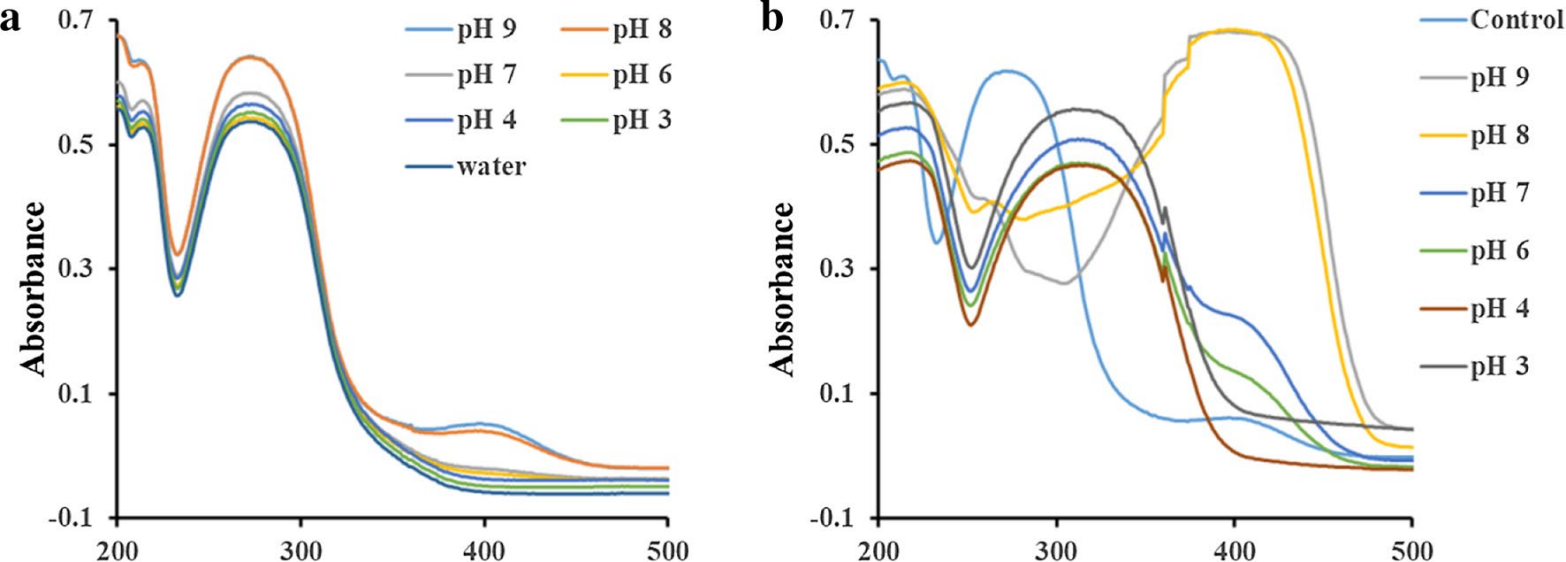
The absorbance spectra of MP in the absence (**a**) and presence (**b**) of Sm-CeO_2_ under different pH condition

Due to the optimal pH for the preparation of polymer, we also investigated the pH effect on the OPH-like activity of Sm-CeO_2_. Firstly, the degradation status of MP was studied without doped nanozyme at different pH. Almost no p-NP was obtained under acidic and neutral condition while few p-NP were yield with characteristic peak at 400 nm under alkaline condition (Fig. 5a). Subsequently, we observed the generation of p-NP from MP with the addition of Sm-CeO_2_ (Fig. 5b), which clearly demonstrated the excellent catalytical activity of nanozyme. The characteristic absorption peak at 310 nm for p-NP was obtained when pH < 8 as well as the peaks at 400 nm when pH > 8. The different maximum absorbance might attribute to the different states of p-NP under acidic and alkaline condition [24, 25]. Since the absorbances of p-NP were reached higher level at both two wavelengths, pH 8 was selected in the further study.

### Establishment of the fluorescent method

**Fig. 6:**
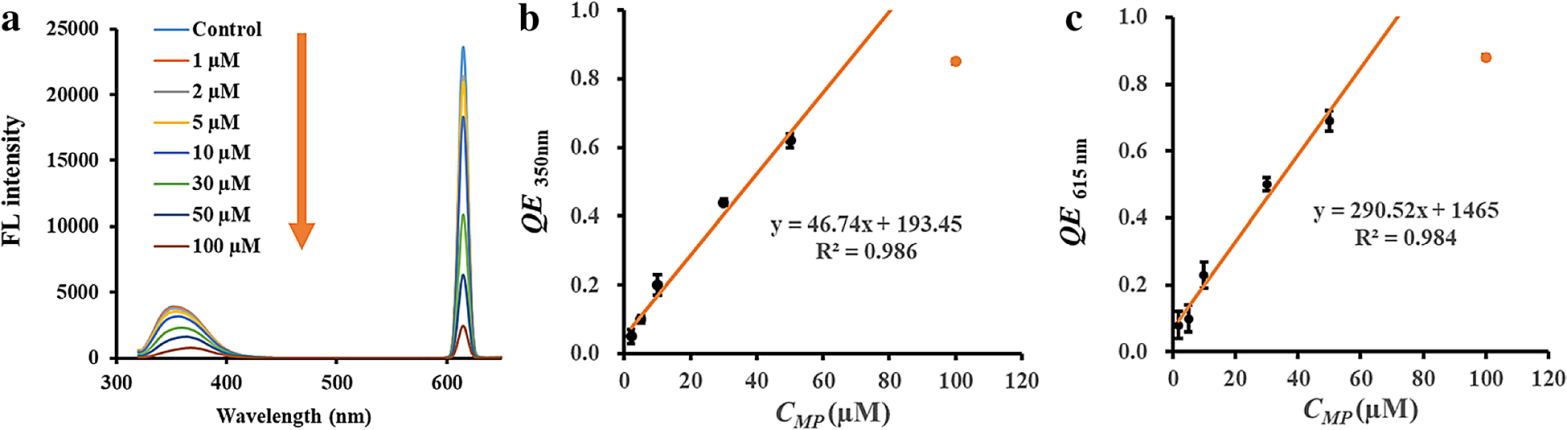
**a** The FL spectra of CFP in the presence of Sm-CeO_2_ and MP ranged from 1 to 100 µmol L^−1^. The dose-dependent relationships between FL quenching effect and MP concentration when emission wavelength was 350 nm (**b**) and 615 nm (**c**)

As reported previously, organophosphate could be hydrolyzed with the aid of OPH [26]. Herein, doped nanozyme Sm-CeO_2_ was discovered with OPH-like activity and applied for MP detection. As expected, p-NP as the degradation product from MP was obtained in the presence of Sm-CeO_2_. It was demonstrated in Fig. 6a, the absorbance peak of p-NP at 400 nm increased gradually with the increasing MP concentration in the presence of Sm-CeO_2_, which clearly indicates Sm-CeO_2_ has OPH-like activity. The CFP were prepared successfully owing to the high affinity of ATP to cerium ions. It was synthesized through a simple self-assembly reaction in one step. Moreover, Tris is used as a cofactor ligand to enhance the luminescence of the polymers. Due to the coordination between cerium ion and the two small molecules (ATP and Tris), the fluorescent emissions were dramatically enhanced, especially for 615 nm (Fig. 3a). The fluorescent peak at 615 nm might attribute to the second harmonic generation peak. Inspired by the feature of CFP, we attempt to detect MP accompanied by turning off the fluorescence. In the present work, p-NP could be hydrolyzed from MP with the help of doped nanozyme. The product interrupted the assemble of CFP and caused fluorescence quenching effect. The amount of MP was observed to exhibit a concentration-dependent effect on the fluorescence decrease, which lays a foundation for detecting MP.

### Fluorescent detection of MP

In the present study, MP was selected to demonstrate the applicability of this detection system for OPP detection. The FL emissions at 350 nm and 615 nm of the polymers were recorded. As displayed in Fig. 6a, the FL intensities at both two wavelengths decreased gradually with the increasing concentration of MP (1–100 μmol L^−1^). In this work, the quenching efficiency (QE) was calculated to measure the MP concentration by Eq. (1).

1 $$QE = 1 - F/F_{0}$$

where *F* and *F*_*0*_ are the FL intensity in the presence and absence of pesticide, respectively. The QE values gradually increased with the increase of MP concentration from 0 to 100 µmol L^−1^. Figure 6b exhibits a linear relationship between the QE _350nm_ (emission at 350 nm) and MP in the range of 2–50 μmol L^−1^ (*R*^*2*^= 0.9860) with a detection limit of 1 µmol L^−1^. To further demonstrate the availability of this detection system, QE _615nm_ (emission at 615 nm) was investigated and display the good linearity with MP in the range of 2–50 μmol L^−1^ (*R*^*2*^= 0.9840), which is comparable with the performance at 350 nm (Fig. 6c).

### Real sample analysis

The present method was further estimated by detecting MP in real samples to study the reliability. The Poria cocos and semen coicis samples were spiked at two levels (10 and 30 μmol L^−1^), and Table 1 shows the recoveries in the range of 73.48–111.46% (n = 3). The results demonstrated the applicability of our method as an effective strategy for MP detection in real sample.

**Table 1:** Recoveries obtained from the detection of methyl-paraoxon in spiked samples

Sample	Detection wavelength	Spiked (µmol L^−1^)	Measured (µmol L^−1^)	Recovery (%)	RSD (%)
*Poria cocos*	350 nm	10	8.83	88.32	12.22
		30	29.97	99.90	1.80
	615 nm	10	11.09	111.09	3.12
		30	33.44	111.46	1.73
Semen coicis	350 nm	10	7.34	73.48	14.66
		30	28.78	95.92	11.00
	615 nm	10	8.43	84.32	9.30
		30	31.73	105.77	7.61

## Conclusions

In this study, fluorescent metal coordination polymers are prepared simply in one step and developed for OPP detection. The proposed method relies on two aspects: one is the excellent OPH-like activity of a new doped nanozyme “Sm-CeO_2_”; the other is the good relationship between the MP concentration and the fluorescent quenching degree of CFPs. As far as we know, it is the first time to use CFP and doped nanozyme to detect OPP, as well as to apply for real sample analysis. The constructed method showed a detection limit of 1.0 µmol L^−1^ (0.06 mg kg^−1^). In summary, the successful application of CFP for MP detection in *Poria cocos* and semen coicis sample shows its great potential without complex equipment and sample preparation process. This newly developed fluorescent assay provides a simple, reliable strategy to detect OPP in Chinese herb samples with extensive compounds.

## Data Availability

The datasets used in this study are available from the corresponding author upon reasonable request.

## Abbreviations

OPP: Organophosphorus pesticide
OPH: Organophosphorus hydrolase
ATP: Adenosine triphosphate
MP: Methyl-paraoxon
Sm-CeO_2_: Samarium doped cerium oxide
p-NP: Para-nitrophenol
CFP: Ce-based fluorescent polymer
QE: Quenching efficiency
